# Comparing g-computation, propensity score-based weighting, and targeted maximum likelihood estimation for analyzing externally controlled trials with both measured and unmeasured confounders: a simulation study

**DOI:** 10.1186/s12874-023-01835-6

**Published:** 2023-01-17

**Authors:** Jinma Ren, Paul Cislo, Joseph C. Cappelleri, Patrick Hlavacek, Marco DiBonaventura

**Affiliations:** 1 Statistical Research & Data Science Center, Pfizer Inc, 500 Arcola Rd, Collegeville, PA 19426 USA; 2 Statistical Research & Data Science Center, Pfizer Inc, New York, NY USA; 3 Statistical Research & Data Science Center, Pfizer Inc, Groton, CT USA; 4 Value & Evidence, Pfizer Inc, New York, NY USA

**Keywords:** External control, G-computation, Propensity Score, Targeted maximum likelihood estimation, Unmeasured confounder, Simulation

## Abstract

**Objectives:**

To have confidence in one's interpretation of treatment effects assessed by comparing trial results to external controls, minimizing bias is a critical step. We sought to investigate different methods for causal inference in simulated data sets with measured and unmeasured confounders.

**Methods:**

The simulated data included three types of outcomes (continuous, binary, and time-to-event), treatment assignment, two measured baseline confounders, and one unmeasured confounding factor. Three scenarios were set to create different intensities of confounding effect (e.g., small and blocked confounding paths, medium and blocked confounding paths, and one large unblocked confounding path for scenario 1 to 3, respectively) caused by the unmeasured confounder. The methods of g-computation (GC), inverse probability of treatment weighting (IPTW), overlap weighting (OW), standardized mortality/morbidity ratio (SMR), and targeted maximum likelihood estimation (TMLE) were used to estimate average treatment effects and reduce potential biases.

**Results:**

The results with the greatest extent of biases were from the raw model that ignored all the potential confounders. In scenario 2, the unmeasured factor indirectly influenced the treatment assignment through a measured controlling factor and led to medium confounding. The methods of GC, IPTW, OW, SMR, and TMLE removed most of bias observed in average treatment effects for all three types of outcomes from the raw model. Similar results were found in scenario 1, but the results tended to be biased in scenario 3. GC had the best performance followed by OW.

**Conclusions:**

The aforesaid methods can be used for causal inference in externally controlled studies when there is no large, unblockable confounding path for an unmeasured confounder. GC and OW are the preferable approaches.

**Supplementary Information:**

The online version contains supplementary material available at 10.1186/s12874-023-01835-6.

## Introduction

In orphan and rare diseases, single-arm trials are common given the impracticability, if not impossibility, of randomized controlled trials [1–3]. In these settings, an external control arm (ECA) can be employed to compare against the single-arm trial to estimate treatment effects, though minimizing potential biases to interpret these effects is critical. Regulatory bodies have issued guidelines for the application of ECA in drug development [4–7]. In practice, the ECAs, their data management, and the assessment of outcomes and confounders should match the prospective trial population and procedures as closely as possible. Even when these criteria are met, the ECA design may inherently introduce further uncertainties to such comparisons due to measured and unmeasured confounders [8]. Therefore, the adjustment for baseline characteristics and potential confounders is one of key steps when an externally control is used as a comparator [9].

Numerous methods have been proposed to handle the potential confounders in ECAs. When individual-level data are available, frequentist approaches may estimate a causal effect using g-computation, propensity score (PS)-based weighting, PS-based stratification, PS-based matching, targeted maximum likelihood estimation (TMLE), etc. [10–14]. Under the circumstances with summary data only, matching-adjusted indirect comparison, simulated treatment outcome, and network meta-analysis may be used to assess the treatment effect [13]. Additionally, Bayesian approaches (e.g., meta-analytic predictive) can also be applied in meta-analysis when combining results from multiple external data sources with either individual or summary data. The adjustments in these methods are based upon measured confounders and incorporated in the analysis. However, the relative performance of these methods in the presence of either unmeasured or unobserved confounders is unclear.

By definition, a confounding variable must be correlated with the outcome and the treatment. The randomization aspect of many studies minimizes the correlations and hence the risk of bias due to confounding. However, when real-word data (RWD) is used to construct an ECA, the benefits of randomization are not possible, and unmeasured or unobserved predictive variables may represent unobserved confounders. Furthermore, the confounder misclassification may lead to another layer of bias when combining trial data and RWD [15]. Although a similar population is selected from RWD with a valid assessment of both outcomes and confounders, this process may not be able to minimize the impact of confounding variables in the way that randomization does. Since we cannot know the confounding effect of unmeasured variables in real-world cases, we wanted to investigate such effects using a simulation study where the effects of the confounder are known. We aimed to compare different methods for estimating average treatment effects (ATEs) in a simulated external control study with individual-level data, which included both measured (included in the model) and unmeasured (excluded from the model) confounders.

## Materials and methods

### Study design

**Fig. 1:**
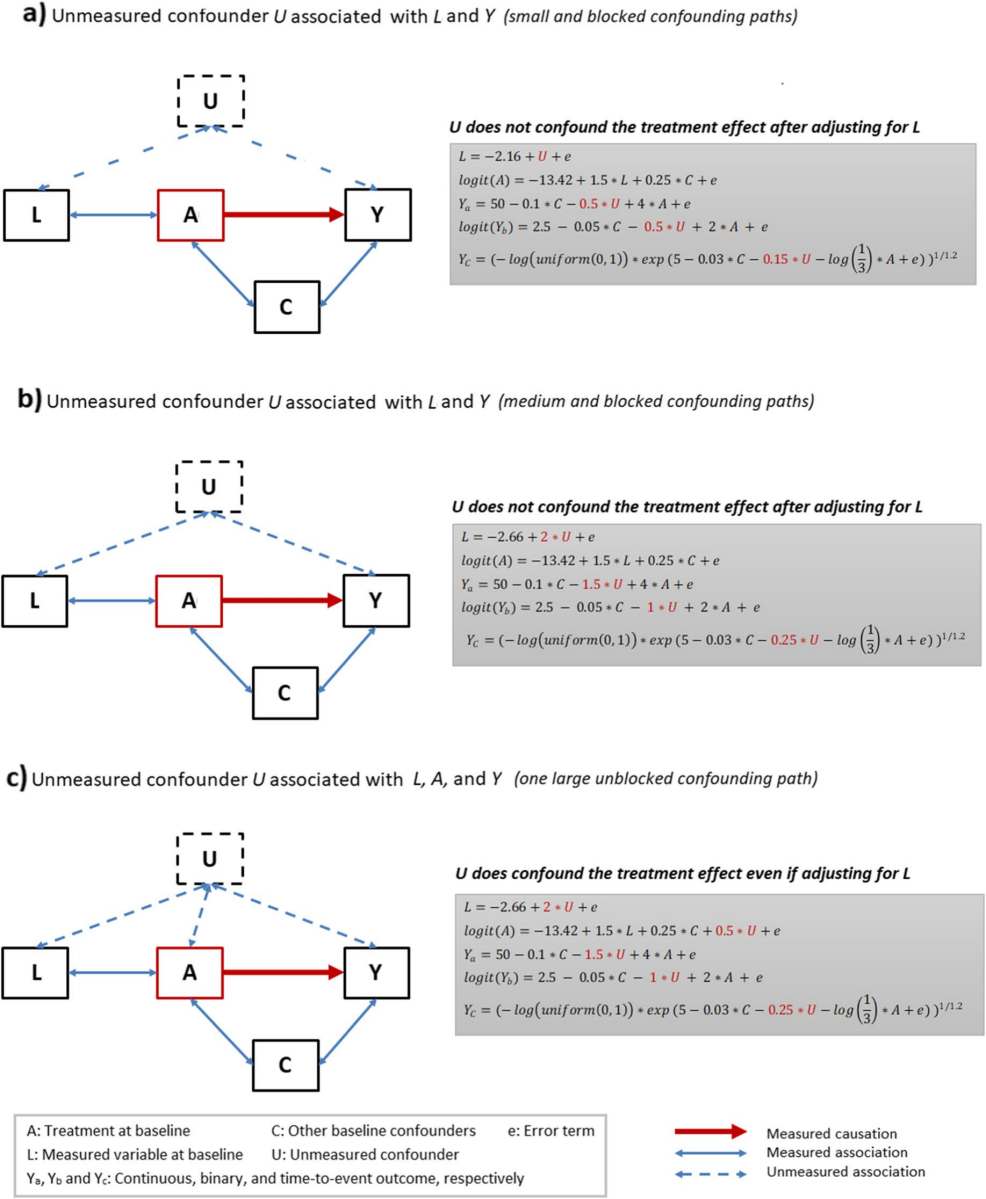
Diagram of measured and unmeasured confounders

We generated a treatment arm (e.g., single-arm trial) and an ECA (e.g., standard of care) in order to simulate an externally controlled study. Three types of outcomes – continuous, binary, and time-to-event endpoints were assessed between two arms. In order to emulate a biased ECA, both measured and unmeasured confounders were added to the data. For each outcome, we explored three scenarios for the relationship between unmeasured confounders and observed variables (Fig. 1).

In scenario 1 & 2, as shown in Fig. 1 (a & b), the baseline covariate *C* was a measured confounder of the relationship between the treatment group assignment *A* and the outcome *Y*. The measured confounder *L* was associated with *A* and the unmeasured confounder *U*. Thus, the unmeasured factor *U* might play an indirect confounding role between the treatment group *A* and the outcome *Y* through the covariate *L*. Meanwhile, we reduced the correlations of *U* with *L* and *Y* in scenario 1 in order to let the confounding effect by *U* be smaller in scenario 1 than scenario 2.

In scenario 3 (Fig. 1c), all relationships in scenario 2 remained the same, but a direct association between *U* and *A* was added. Therefore, the unmeasured factor *U* might play a stronger confounding role between the treatment *A* and the outcome *Y*. While *U* was technically an unmeasured confounder in all three scenarios, we expected it did not confound the treatment effect conditional on the measured confounder *L* in scenario 1 and 2. On the other hand, the direct association between *U* and the treatment group assignment *A* added in scenario 3 could lead to confounded estimates even if adjusting for *L* as there was an open path from treatment to the outcome through it.

A set of random samples were drawn from a simulated target population for each scenario. For each generated sample, g-computation, PS-based weighting, e.g., inverse probability of treatment weighting (IPTW), standardized mortality/morbidity ratio (SMR), and overlap weighting (OW), and targeted maximum likelihood estimation (TMLE) were used to estimate treatment effects based on observed outcomes and measured confounders which were incorporated in the analytic model.

### Simulated dataset

We generated a data set with 20,000 individuals, which was considered as a target population. In this data set, variable *U* (deemed as an unmeasured confounder) included random numbers from the normal distribution (mean 0.5, variance 1); Variable *C* was a baseline characteristic from the normal distribution (mean 50, variance 50); variable *L* was a binary covariate, which was a logit function of *U*; variable A was the treatment assignment (0 = ECA,1 = Trial arm), which was a logit function of *L* and *C* in scenario 1 & 2 and a logit function of *L*, *C* and *U* in scenario 3; Variables *Y*_*a*_, *Y*_*b*_, and *Y*_*c*_ were continuous, binary, and time-to-event outcomes, respectively. These outcomes were a corresponding function of *A*, *C*, and *U*. In addition, variable *D* indicated the event (e.g., 0 = alive, 1 = dead) for the time-to-event outcome *Y*_*c*_.

From this target population, we randomly drew 3000 samples. Each sample contained 200 observations (i.e. ‘individuals’) and were randomly drawn from the target population. Also, we drew another 3000 samples with 100 individuals per sample in a similar fashion, in order to further validate our results given a different sample size.

### True effect

For assessing the various methods that obtained estimates based on samples, the true effect of treatment in the population is needed. Population level ATEs were obtained by applying regression models to estimate marginal effects in the full dataset (n = 20,000), including both measured and unmeasured confounders in the analyses. The ATEs of *Y*_*a*_, *Y*_*b*_, and *Y*_*c*_ were assessed by mean difference, log odds ratio, and log hazard ratio, respectively.

### Methods for minimizing potential biases

This study explores g-computation, PS-based weighting, and TMLE methods that can be used to handle individual-level data for reducing potential biases in externally controlled studies.

#### G-computation

G-computation is one of Robins’ g methods [16]. It is used for modelling the joint density of the observed data to generate potential outcomes under different exposure scenarios [10]. First, we used the observed data (variables A, C, L) to build the outcome regression models, such as linear regression, logistic regression, and Cox proportional hazards regression for ${\widehat{Y}}_{a}$, ${\widehat{Y}}_{b}$, and ${\widehat{Y}}_{c}$, respectively. Then, the counterfactual outcomes for each individual were estimated based on the outcome regression models by assuming all individuals received the trial treatment and the standard of care at the same time. Finally, the estimated ATE for each outcome was calculated, which was the difference between the two averages of counterfactual outcomes. The R package “RISCA” was used to implement g-computation for estimating ATE for binary and time-to-event outcomes [12].

#### PS-based weighting

One of common PS-based weighting method is IPTW [17]. SMR and OW methods can be also used to create a weight for minimizing the potential biases in a causal effect estimation [14, 18, 19]. We used logistic regression to predict the probability of treatment assignment (*p*_i_, propensity score for each individual) given the observed predictors of *C* and *L*. Then, a weight for each individual was calculated according to its corresponding weighting method. The weights were 1/*p*_i_, 1, and 1-*p*_i_ in the trial treatment group, whereas they were 1/(1-*p*_i_), *p*_i_ /(1-*p*_i_), and *p*_i_ in the ECA group when using IPTW, SMR, and OW, respectively. The PS-based weight was further applied in linear regression, logistic regression, and Cox proportional hazards regression for estimating the ATEs of ${\widehat{Y}}_{a}$, ${\widehat{Y}}_{b}$, and ${\widehat{Y}}_{c}$, respectively.

##### TMLE

The TMLE method puts g-computation and PS-based weighting together, which is also considered as doubly robust estimation [11, 20, 21]. In brief, ATE estimation with TMLE begins with estimation of the conditional expectation of the outcome given the exposure and covariates, *E(Y|A, C, L)*. This estimate of *E(Y|A, C, L)* is used to generate predicted values of the outcome for both exposure levels (e.g., the pair of potential outcomes). It is also called the initial counterfactual outcomes for each individual using outcome regression model, which is similar to those steps in g-computation. Next, the “targeting” step involves estimation of the exposure mechanism, *P(A* = *1|C, L)*, which is then used to update the initial estimate of *E(Y|A, C, L)* through the use of a predefined working regression model. In this step, the PS-based clever covariate $H\left(A, C,L\right)=\frac{I\left(A=1\right)}{P(A=1|C,L)}-\frac{I\left(A=0\right)}{P(A=0|C,L)}$ is included in the equation $logit\left({\widehat{Y}}^{*}\right)=logit\left(\widehat{Y}\right) + \in *H$ in order to estimate the fluctuation parameter ($\in$) that provides the information about how much to change, or fluctuate, our initial outcome estimate.

Last, the estimated ATE for each outcome can be calculated as follows:

$$\widehat{ATE}=\frac{1}{n}{\sum }_{i=1}^{n}({\widehat{Y}}_{1}^{*}-{\widehat{Y}}_{0}^{*})$$

The TMLE method was conducted using R package “ltmle” with the default machine learning algorithms. Since the package “ltmle” cannot directly provide hazards ratio (HR) for time-to-event outcome (${\widehat{Y}}_{c}$), relative risk (RR) at the primary time point (e.g. median survival of all individuals) was estimated first, then converted into HR using the following equation:

$$\mathrm{HR}=\frac{\mathrm{log}(1-\mathrm{d}*\mathrm{RR})}{\mathrm{log}(1-\mathrm{d})}$$

where RR is the relative risk, HR is the hazard ratio, and d is the death rate for reference group (e.g., d = 0.4) [22].

### Model comparison

All statistical analyses were conducted in RStudio (version 1.4.1717) and R (version 4.0.4). In addition to the aforementioned methods, we also conducted the analysis using a raw model, which directly estimated the treatment effects without any adjustment. In order to assess these methods, we compared the true effect with the results (point estimate and 95% confidence interval) from each model, and calculated the bias, root mean squared error (RMSE) along with coverage and width of 95% confidence interval for the treatment effects. The bias was defined as the average difference between the true value (simulated) and its estimate across the simulation replicates using the original scale for the continuous outcome and the log-transformed scale for the binary and time-to-event outcomes, such as log(HR). RMSE was the square root of the mean squared error (MSE) that was the average squared difference between the true value and its estimate across the simulation replicates. Coverage was the proportion of times the 95% confidence interval of the estimate contained the true value. Width was the average difference between the upper and lower bounds of 95% confidence interval of estimate.

## Results

### Summary of potential confounders

As shown in Table 1, the distribution of three potential confounders (*U*, *C*, and *L*) were considerably different between two treatment arms with a range of standardized mean difference from 0.08 to 0.88.

**Table 1:** Summary of potential confounders between treatment groups

Scenario/Variable	Control (A = 0)	Treatment (A = 1)	Standardized mean difference
**Scenario 1**			
U (mean ± SD)	0.45 ± 0.99	0.56 ± 1.01	0.08
C (mean ± SD)	46.97 ± 6.12	54.39 ± 6.06	0.86
L (%)	17.96	33.98	0.42
**Scenario 2**			
U (mean ± SD)	0.40 ± 0.97	0.62 ± 1.02	0.16
C (mean ± SD)	46.74 ± 6.08	54.24 ± 5.93	0.88
L (%)	20.63	40.38	0.44
**Scenario 3**			
U (mean ± SD)	0.27 ± 0.95	0.76 ± 1.00	0.36
C (mean ± SD)	46.66 ± 6.14	53.84 ± 6.05	0.83
L (%)	17.38	42.92	0.55

Scenario 1, 2 & 3 stands for small and blocked confounding paths, medium and blocked confounding paths, and one large unblocked confounding path, respectively. The standardized mean difference expresses the size of the intervention effect in each study relative to the variability observed in that study

*SD* Standard deviation, *U* Unmeasured confounder, *C & L* Measured confounders

The unmeasured factor *U* was associated with the outcomes in the three scenarios. The values of Pearson’s correlation (*r*) of outcomes (*Y*_*a*_*, Y*_*b*_, and *Y*_*c*_) with *U* were -0.15, -0.12, and -0.07 in scenario 1, respectively. These correlation values were increased accordingly in scenario 2, especially for *Y*_*a*_ (-0.44) followed by *Y*_*b*_ (-0.23), and *Y*_*c*_ (-0.12), which were similar with scenario 3.

The correlation (*r*) between the unmeasured factor *U* and the treatment assignment *A* was 0.06 and 0.11 in scenario 1 & 2, respectively, and it became much stronger (*r* = 0.24) in scenario 3 after adding a direct association between them. The treatment assignment *A* was also influenced by other baseline factors *C* (*r* = 0.51, 0.53 and 0.51, respectively) and *L* (*r* = 0.18, 0.22 and 0.28, respectively) in the three scenarios.

The odds ratio between the unmeasured confounder *U* and the treatment assignment *A* adjusting for the measured factor *L* was 0.99, 0.99, and 1.34 in scenario 1, 2, and 3, respectively. It indicated that the measured factor *L*, as an alternative variable of unmeasured factor *U*, could not block the confounding path (*A ← U → Y*) in scenario 3.

### Estimated ATE for continuous endpoint

**Fig. 2:**
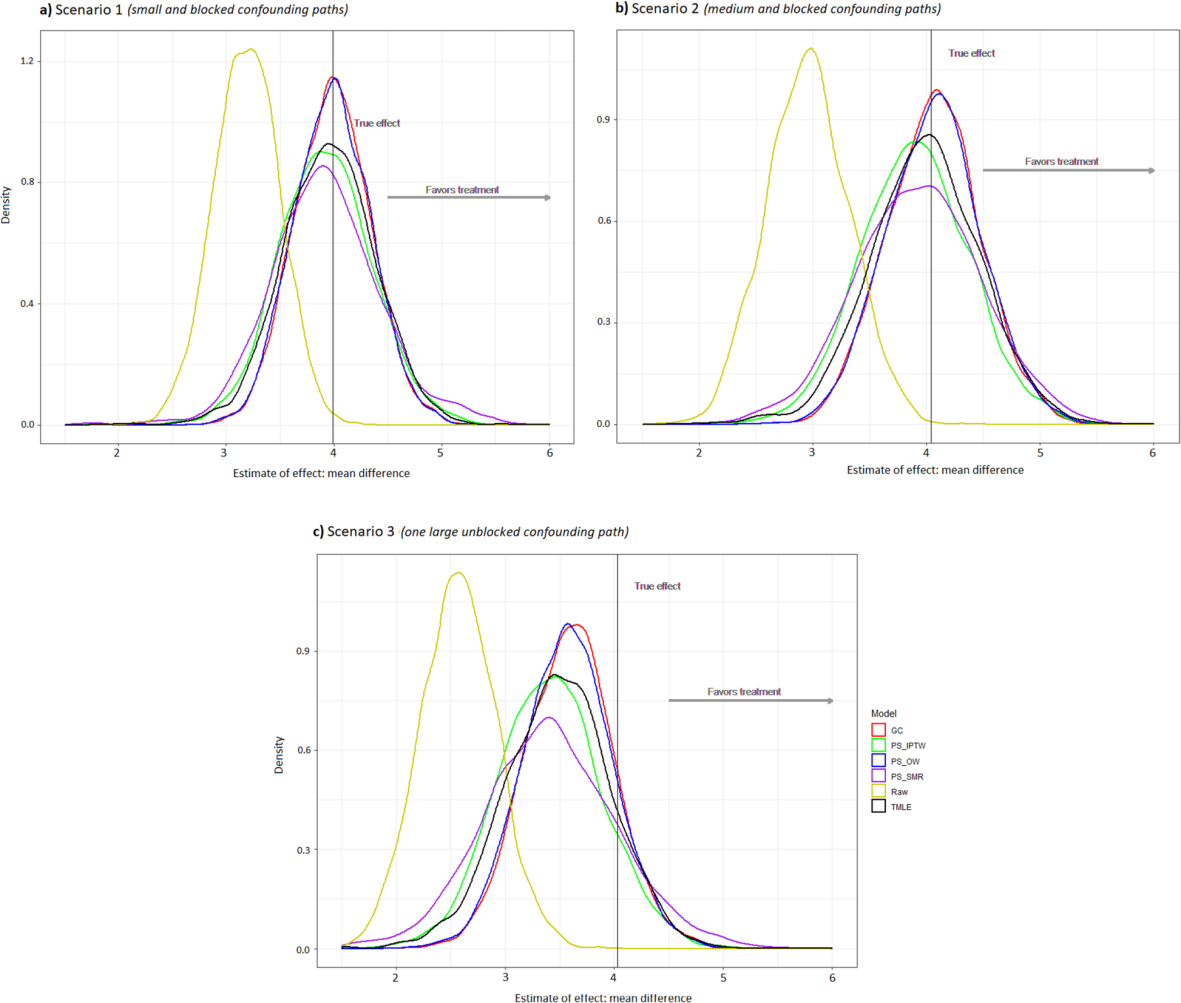
Density distribution of treatment effect estimates by different methods for a continuous endpoint (*n* = 200). GC, g-computation; PS-, propensity score-based; IPTW, inverse probability of treatment weighting; SMR, standardized mortality or morbidity ratio; OW, overlap weighting; TMLE, targeted maximum likelihood estimation

As depicted in Fig. 2, the center of estimated ATE distribution (mean ATE = 3.19) for continuous endpoint from the raw model obviously deviated from the true effect (mean difference = 3.99) in scenario 1. The poor performance of raw model was also reflected in the larger RMSE (0.86) and smaller coverage (0.26) compared with other methods (Table 2—Outcome *Y*_*a*_). On the contrast, the mean estimated ATEs for continuous endpoint from g-computation, IPTW, OW, SMR, and TMLE were 3.99, 3.92, 3.98, 3.92, and 3.95, respectively, which were close to the true effect. Among them, g-computation had the smallest RMSE (0.351) followed by OW (RMSE = 0.354), and g-computation had the greatest coverage of 0.95.

**Table 2:** Performance of different methods for estimating the treatment effects (*n* = 200)

Outcome/Method	Scenario 1 (small and blocked confounding paths)				Scenario 2 (medium and blocked confounding paths)				Scenario 3 (one large unblocked confounding path)			
	Bias	RMSE	Coverage	Width	Bias	RMSE	Coverage	Width	Bias	RMSE	Coverage	Width
**Outcome *Y***_***a***_												
Raw	-0.800	0.856	0.264	1.205	-1.086	1.145	0.164	1.429	-1.461	1.502	0.016	1.393
GC	-0.001	0.351	0.950	1.376	0.022	0.408	0.948	1.617	-0.443	0.600	0.809	1.613
PS_IPTW	-0.066	0.447	0.831	1.190	-0.138	0.500	0.843	1.417	-0.618	0.784	0.548	1.382
PS_OW	-0.006	0.354	0.899	1.164	0.024	0.416	0.902	1.384	-0.458	0.616	0.696	1.356
PS_SMR	-0.072	0.521	0.766	1.169	-0.111	0.568	0.778	1.392	-0.625	0.878	0.513	1.370
TMLE	-0.039	0.429	0.928	1.514	-0.054	0.488	0.922	1.726	-0.548	0.731	0.734	1.724
**Outcome *Y***_***b***_												
Raw	-0.267	0.405	0.854	1.208	-0.326	0.443	0.796	1.177	-0.432	0.524	0.687	1.157
GC	0.014	0.355	0.938	1.367	0.014	0.349	0.934	1.328	-0.133	0.376	0.917	1.332
PS_IPTW	0.037	0.443	0.836	1.223	0.000	0.430	0.848	1.196	-0.179	0.470	0.794	1.176
PS_OW	0.037	0.381	0.894	1.211	0.041	0.372	0.891	1.192	-0.126	0.394	0.867	1.174
PS_SMR	0.000	0.500	0.777	1.200	-0.028	0.497	0.773	1.183	-0.222	0.573	0.703	1.169
TMLE	0.036	0.432	0.974	1.885	0.015	0.423	0.974	1.869	-0.166	0.462	0.958	1.857
**Outcome *Y***_***c***_												
Raw	0.181	0.248	0.792	0.662	0.201	0.261	0.773	0.658	0.237	0.290	0.695	0.650
GC	-0.002	0.190	0.926	0.717	-0.006	0.189	0.932	0.725	0.048	0.196	0.922	0.724
PS_IPTW	0.017	0.237	0.934	0.865	0.015	0.241	0.937	0.867	0.059	0.243	0.923	0.852
PS_OW	-0.026	0.208	0.948	0.796	-0.035	0.212	0.941	0.808	0.023	0.211	0.942	0.806
PS_SMR	-0.015	0.234	0.929	0.838	-0.031	0.238	0.922	0.847	0.033	0.245	0.925	0.862
TMLE	0.146	0.317	0.901	1.191	0.135	0.302	0.908	1.173	0.170	0.312	0.902	0.139

Bias was the average difference between the true value (simulated) and its estimate across the simulation replicates using the original scale for the continuous outcome and the log-transformed scale for the binary and time-to-event outcomes, such as log (HR). RMSE was the square root of the mean squared error (MSE) that is the average squared difference between the true value and its estimate across the simulation replicates. Coverage was the proportion of times the 95% confidence interval of the estimate contained the true value. Width was the average difference between the upper and lower bounds of 95% confidence interval of estimate

*GC* G-computation, *RMSE* Root mean squared error, *PS*- propensity score-based, *IPTW* Inverse probability of treatment weighting, *SMR* Standardized mortality or morbidity ratio, *OW* Overlap weighting, *TMLE* Targeted maximum likelihood estimation

In scenario 2, the mean estimated ATE (2.95) for continuous endpoint from the raw model shifted further away from the true effect (4.04). The mean estimates from g-computation, IPTW, OW, SMR, and TMLE were 4.06, 3.90, 4.06, 3.93, and 3.99, respectively, which were still acceptably close to the true effect. However, the mean estimates from these adjusted methods tended to be more biased in scenario 3 compared with scenario 2 (e.g., RMSE for g-computation was 0.60 vs 0.41).

### Estimated ATE for binary endpoint

**Fig. 3:**
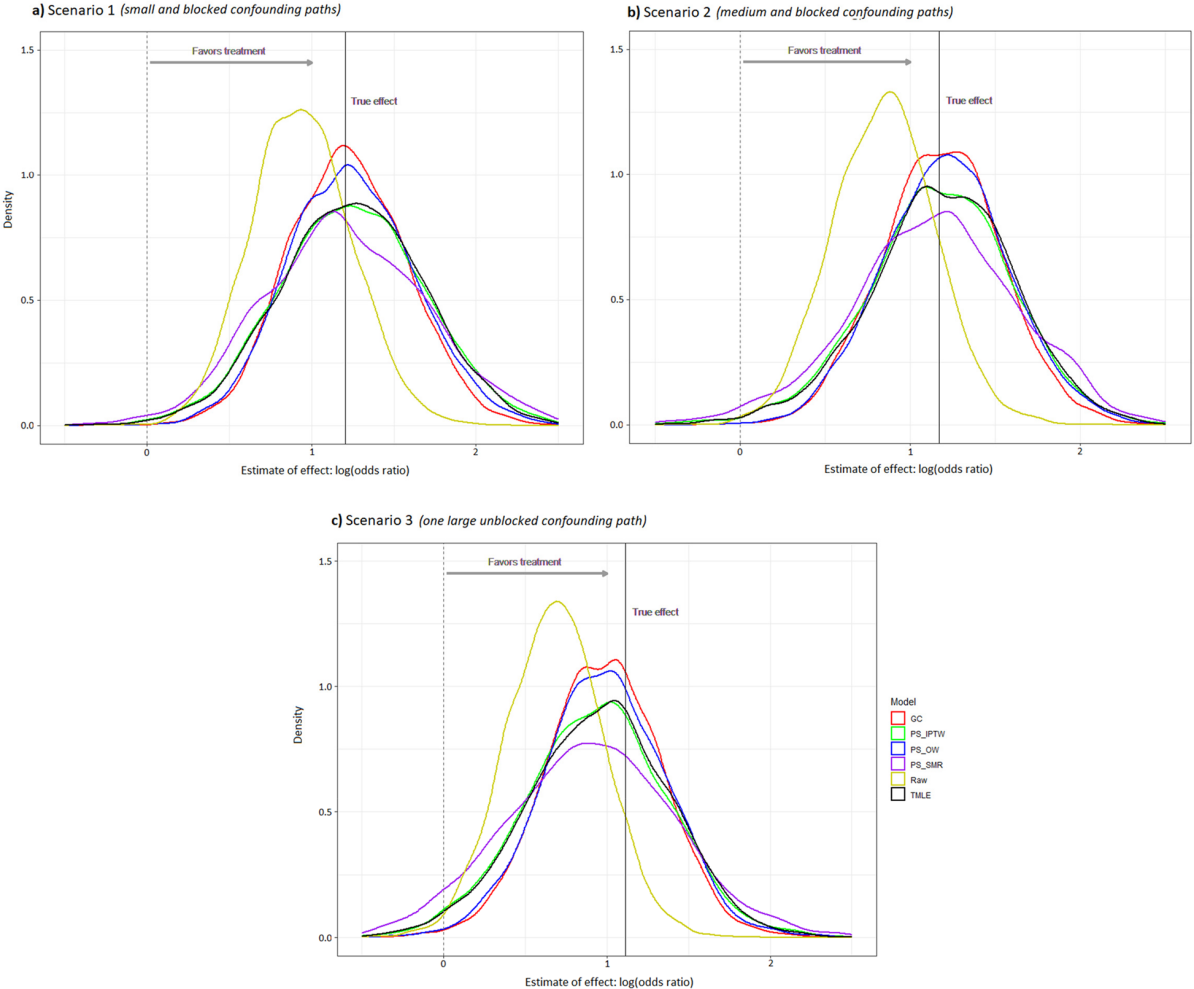
Density distribution of treatment effect estimates by different methods for a binary endpoint (*n* = 200). GC, g-computation; PS-, propensity score-based; IPTW, inverse probability of treatment weighting; SMR, standardized mortality or morbidity ratio; OW, overlap weighting; TMLE, targeted maximum likelihood estimation

Similar to the results for the continuous endpoint, the mean estimated ATE (0.94) for binary endpoint from the raw model obviously deviated from the true effect (log odds ratio = 1.21) in scenario 1, and the deviation was even larger in scenario 2 & 3. As shown in Fig. 3, the mean estimated ATEs for binary endpoint from g-computation, IPTW, OW, SMR, and TMLE approximated to the true effect in scenario 1 (1.22, 1.24, 1.24, 1.21, and 1.24 versus 1.21, respectively) and in scenario 2 (1.19, 1.17, 1.21, 1.14, and 1.19 versus 1.17, respectively), but they deviated a little from the true effect in scenario 3 (0.98, 0.93, 0.99, 0.89, and 0.95 versus 1.11, respectively). Compared with other methods (Table 2—Outcome *Y*_*b*_), g-computation still had the smallest RMSE (e.g., 0.35 in scenario 2) followed by OW (e.g., 0.37 in scenario 2) along with a reasonable coverage (e.g., 0.93 in scenario 2).

### Estimated ATE for time-to-event endpoint

**Fig. 4:**
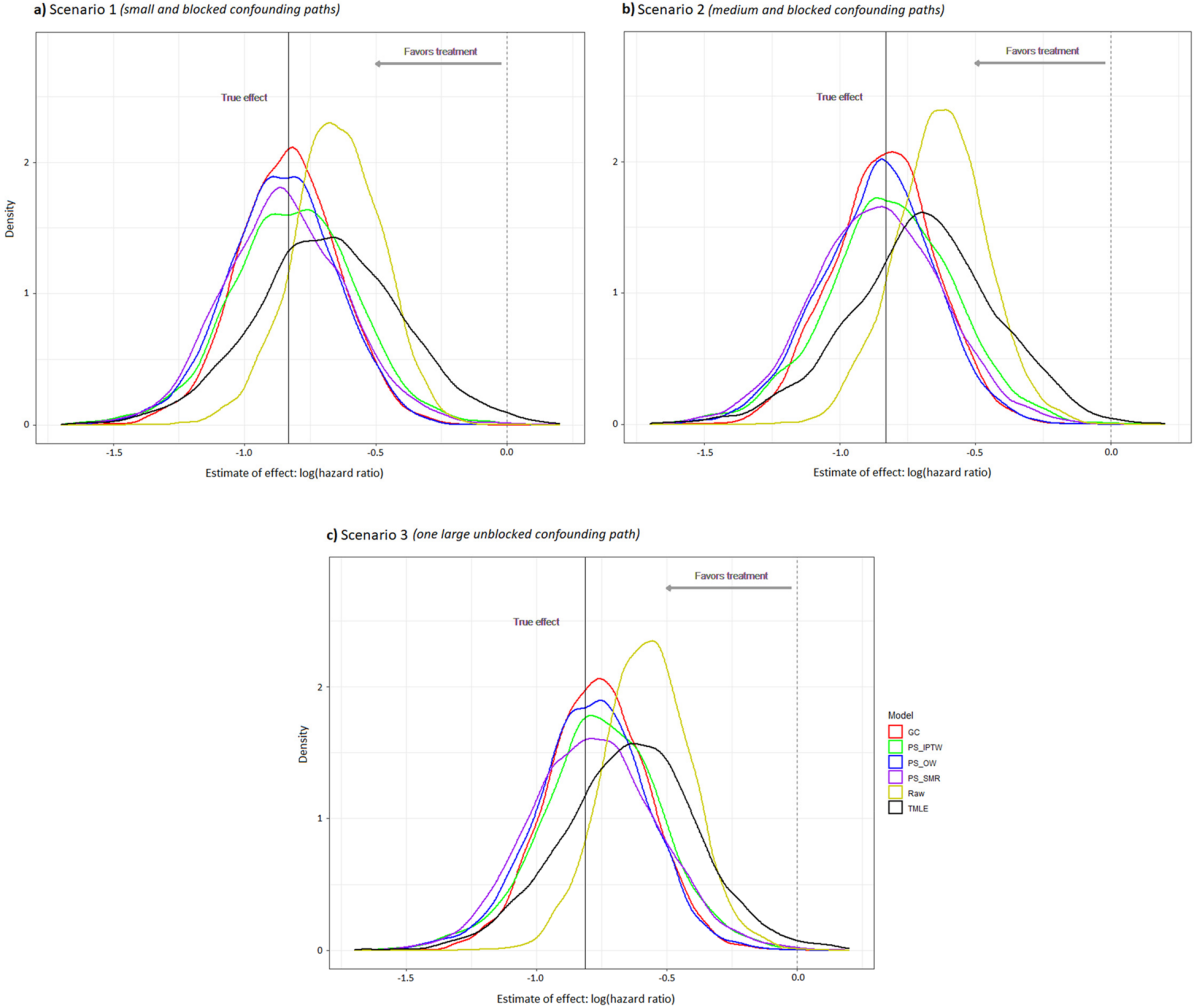
Density distribution of treatment effect estimates by different methods for a time-to-event endpoint (*n* = 200). GC, g-computation; PS-, propensity score-based; IPTW, inverse probability of treatment weighting; SMR, standardized mortality or morbidity ratio; OW, overlap weighting; TMLE, targeted maximum likelihood estimation

The mean estimated ATEs for time-to-event endpoint from the raw model also considerably differed from the true effects in all scenarios (e.g., log hazard ratio = -0.65 versus -0.83 in scenario 1). The mean estimated ATEs for time-to-event endpoint from g-computation, IPTW, OW, SMR, and TMLE in scenario 1 were -0.83, -0.81, -0.86, -0.85, and -0.68, respectively, which closely approximated to the true effect except the estimate by TMLE with a conversion from RR to HR had an obvious deviation (-0.68 versus -0.83). As shown in Fig. 4, the similar results were observed in scenario 2 & 3. The superior performance of g-computation, IPTW, OW, and SMR was also demonstrated in Table 2 (Outcome *Y*_*c*_), and g-computation had the smallest RMSE in all three scenarios (e.g., 0.19 for scenario 1) compared with other methods.

### Sensitivity analysis

When the sample size was reduced from 200 to 100, we found similar results in terms of reduction in bias associated with each method, but we observed increase of variance, which led to a larger MSEs and wider confidence intervals (supplementary file S1).

## Discussion

By design, both the unmeasured confounder *U* and the measured covariates (*C* and *L*) played a role of potential confounders when we investigated the treatment effect in the simulated data. The raw or unadjusted (observed) model that ignored all the potential confounders produced results which were obviously biased, deviating greatly from the known treatment effect. When including the measured covariates in the model only, the methods of g-computation, PS-based weighting (IPTW, OW and SMR), and TMLE produced results that were less biased than the raw analysis approach in the three scenarios. Theoretically, the bias due to unmeasured confounder cannot be completely corrected unless an alternative variable is measured and included in the analysis.

In scenario 1 & 2, the unmeasured confounder *U* indirectly influenced the treatment assignment through the covariate *L*, and it had a different strength of association with each outcome (*Y*_*a*_ > *Y*_*b*_ > *Y*_*c*_). Thus, we found that the unmeasured confounder had the strongest confounding effect on outcome *Y*_*a*_ compared with other outcomes (Fig. 2,3,4). Under this circumstance, the methods of g-computation, PS-based weighting, and TMLE successfully corrected for most of bias when we investigate the treatment effect based upon the measured data. Here, the covariate *L* played a role of alternative variable for the unmeasured confounder *U*, so these methods that incorporated the covariate *L* worked well.

After adding an additional relationship between *U* and treatment assignment, the unmeasured confounder showed a stronger confounding effect in scenario 3 than scenario 2, based upon the standardized mean difference (0.36 vs 0.16). It can be mainly explained by the reason that the covariate *L* in scenario 3 was not a good alternative variable for the unmeasured confounder *U* anymore because *U* was also directly associated with treatment assignment. Thus, it is not surprising to see more biases in scenario 3 compared to the other two scenarios. Furthermore, the unmeasured confounder had a relatively strong correlation with outcome *Y*_*a*_. That may be the reason why the results for outcome *Y*_*a*_ by all methods were tended to be more biased in scenario 3 than scenario 2 (Fig. 2). The extent of the bias became relatively small for outcome *Y*_*b*_ and even negligible for outcome *Y*_*c*_ because their associations with the unmeasured confounder were not as strong as outcome *Y*_*a*_.

Although the same predictors were used for modeling, the g-computation had the smallest RSMEs in most of settings of this simulation study (Table 2), which is consistent with the other recent simulation study [12]. Currently, the PS-based approach is still relatively more predominant relative to G-computation for a handful of pragmatic reasons. First, the PS-based approach is easy to understand although it also requires those assumptions that g-computation needs, such as counterfactual consistency, exchangeability, and positivity [10]. Second, the variance formula is explicit for the PS-based approach, but g-computation usually needs a bootstrapping or simulation to obtain the variance [12, 23]. Third, several tutorials on PS-based approach and its successful applications in research and regulatory approvals can be found [24–28]. However, these reasons should not stop g-computation from becoming another popular alternative approach to minimize potential biases in the future drug development.

TMLE is a doubly robust maximum-likelihood-based approach that includes a secondary "targeting" step that optimizes the bias-variance tradeoff for the target parameter [11]. Unsurprisingly, the RSMEs for TMLE were between those for by g-computation and IPTW in our simulation study, since TMLE is considered as a combination of g-computation and PS-based weighting. Undoubtedly, TMLE has its merit that the doubly robust property helps TMLE even against significant model misspecification arising from an omitted confounder in either the exposure or outcome regressions [11]. However, TMLE couldn’t demonstrate its advantage in our simulation study because the unmeasured confounder was omitted in both the exposure and outcome regressions, which is not uncommon in real-world data. In addition, we observed more biases in the estimates of treatment effect for the time-to-event outcome by TMLE compared with other adjusted methods. One explanation for the deviation might be the use of conversion from RR to HR in our study, because the current R packages (e.g., “MOSS”, “survtmle”, “ltmle”) are not able to provide a HR directly. The lack of HR statistics in the current statistical package might be one of hurdles for the application of TMLE in practice. On the other hand, the use of HR is not indispensable in survival analysis, and survival curves are more informative than HRs although the HR is the main, and often the only, effect measure reported in many epidemiological studies [29].

Among the three PS-based weighting methods, OW had the best performance in this simulation. Both IPTW and SMR use the propensity score as a part of denominator for calculating the weight, but OW does not. The weight using reciprocal of propensity score could be greatly amplified when the propensity score is very small or large (e.g. 0.01 or 0.99). Therefore, OW may be less sensitive to the extreme values of propensity score, which might lead to a smaller RSME for estimating the treatment effect compared with IPTW and SMR [19]. Furthermore, the target populations of three PS-based weighting methods may be interpreted differently. The estimates by IPTW, SMR, and OW could be considered as the average treatment effect in the population, the treated, and the overlap, respectively. In a sensitivity analysis with some heterogeneity of treatment effect (supplementary file S2), we further verified that IPTW, SMR, and OW are not interchangeable methods except under constant treatment effects. Both g-computation and TMLE can also be implemented to estimate the treatment effect for different target population, such as average treatment effect in the entire population (ATE), treatment effect of the treated (ATT), and treatment effect of the untreated (ATU).

One of merits in this study is that we explored some scenarios which the previous studies had not investigated, such as three types of outcomes along with different confounding effects (small and blocked confounding paths, medium and blocked confounding paths, and one large unblocked confounding path) caused by an unmeasured confounder [11, 12]. However, it might be worth exploring more scenarios in the future studies. For instance, what if ECA studies have longitudinal data with time-varying measured and unmeasured confounders? In addition, since we targeted on the marginal treatment effect, our simulation did not provide the conditional treatment effect, which is usually estimated by the multivariable regression model adjusting for confounders [30]. Finally, our simulation did not include other doubly robust methods (e.g., augmented-IPTW) because their doubly robust property is similar to TMLE [11, 31–33].

## Conclusion

In externally controlled studies, the methods of g-computation, PS-based weighting (IPTW, OW and SMR), and TMLE can be used to minimize the biases due to measured and unmeasured confounders. However, the extent of bias reduction by these methods depends on the technique used and the existence of an unblockable confounding path for an unmeasured confounder. Our findings in this simulation study suggest that g-computation and OW are the preferable alternative approaches producing relatively unbiased estimates, especially when there is no large, unblocked confounding path for an unmeasured confounder.

## Supplementary Information

**Additional file 1:** **Supplementary file S1.** Sensitivity analysis with a different sample size.

**Additional file 2:** **Supplementary file S2.** Sensitivity analysis with a different treatment effect.

**Additional file 3:** **Supplementary file S3.** R programming example for the simulation.

## Data Availability

The example of R codes for data simulation and analysis can be found in the supplementary file S3. Please contact the corresponding author (Jinma Ren, jinma.ren@pfizer.com) if there are any questions on the data simulation.
