# Supplementary material for: Comparing g-computation, propensity score-based weighting, and targeted maximum likelihood estimation for analyzing externally controlled trials with both measured and unmeasured confounders: a simulation study
Source: BMC Med Res Methodol. 2023 Jan 17;23:18. doi: 10.1186/s12874-023-01835-6 (PMC9843888; doi:10.1186/s12874-023-01835-6)
Supplement: Supplementary file 1 — Additional file 1: Supplementary file S1. Sensitivity analysis with a different sample size. [file 12874_2023_1835_MOESM1_ESM.pdf]

Supplementary file S1: Performance of different methods for estimating the treatment effects (n=100)

Outcome /Method	Scenario 1 *(small and blocked confounding paths)*				Scenario 2 *(medium and blocked confounding paths)*				Scenario 3 *(one large unblocked confounding path)*			
	Bias	RMSE	Coverage	Width	Bias	RMSE	Coverage	Width	Bias	RMSE	Coverage	Width
**Outcome Y_a**												
Raw	-0.807	0.913	0.545	1.711	-1.116	1.235	0.437	2.029	-1.482	1.564	0.168	1.978
GC	-0.011	0.499	0.950	1.968	0.001	0.605	0.934	2.301	-0.456	0.747	0.862	2.296
PS_IPTW	-0.105	0.628	0.828	1.678	-0.176	0.739	0.824	1.997	-0.645	0.955	0.665	1.950
PS_OW	-0.016	0.508	0.894	1.642	-0.002	0.617	0.883	1.950	-0.476	0.767	0.776	1.910
PS_SMR	-0.097	0.701	0.776	1.642	-0.166	0.817	0.773	1.954	-0.666	1.094	0.604	1.919
TMLE	-0.064	0.601	0.905	2.009	-0.088	0.709	0.886	2.268	-0.568	0.896	0.769	2.259
**Outcome Y_b**												
Raw	-0.259	0.514	0.899	1.737	-0.329	0.545	0.874	1.688	-0.434	0.605	0.819	1.658
GC	0.023	0.513	0.931	2.031	0.020	0.505	0.920	1.936	-0.128	0.515	0.923	1.931
PS_IPTW	0.041	0.636	0.855	1.767	0.015	0.620	0.851	1.728	-0.176	0.646	0.816	1.696
PS_OW	0.061	0.561	0.887	1.747	0.063	0.557	0.889	1.717	-0.110	0.550	0.877	1.689
PS_SMR	0.008	0.713	0.796	1.737	-0.028	0.730	0.776	1.717	-0.215	0.796	0.728	1.700
TMLE	0.039	0.618	0.973	2.753	0.024	0.608	0.975	2.722	-0.168	0.628	0.965	2.695
**Outcome Y_c**												
Raw	0.170	0.297	0.892	0.952	0.203	0.317	0.848	0.942	0.240	0.343	0.801	0.931
GC	-0.011	0.272	0.926	1.057	-0.011	0.276	0.922	1.062	0.048	0.288	0.924	0.061
PS_IPTW	0.011	0.340	0.919	1.170	0.011	0.338	0.923	1.171	0.065	0.343	0.905	0.152
PS_OW	-0.040	0.305	0.939	1.135	-0.042	0.310	0.940	1.150	0.019	0.315	0.930	0.145
PS_SMR	-0.016	0.327	0.923	1.138	-0.022	0.338	0.919	1.149	0.051	0.358	0.901	0.154
TMLE	0.130	0.419	0.920	1.749	0.131	0.398	0.934	1.711	0.180	0.410	0.924	0.654

GC, g-computation; RMSE, root mean squared error; PS-, propensity score-based; IPTW, inverse probability of treatment weighting; SMR, standardized mortality or morbidity ratio; OW, overlap weighting; TMLE, targeted maximum likelihood estimation. Bias was the average difference between the true value (simulated) and its estimate across the simulation replicates. RMSE was the square root of the mean squared error (MSE) that is the average squared difference between the true value and its estimate across the simulation replicates. Coverage was the proportion of times the 95% confidence interval of the estimate contained the true value. Width was the average difference between the upper and lower bounds of 95% confidence interval of estimate.
