# Supplementary material for: Comparing g-computation, propensity score-based weighting, and targeted maximum likelihood estimation for analyzing externally controlled trials with both measured and unmeasured confounders: a simulation study
Source: BMC Med Res Methodol. 2023 Jan 17;23:18. doi: 10.1186/s12874-023-01835-6 (PMC9843888; doi:10.1186/s12874-023-01835-6)
Supplement: Supplementary file 2 — Additional file 2: Supplementary file S2. Sensitivity analysis with a different treatment effect. [file 12874_2023_1835_MOESM2_ESM.pdf]

Supplementary file S2.

In this sensitivity analysis, we changed the magnitude of treatment effect (A) for the binary outcome (Y_b) in Scenario 3 (one large unblocked confounding path) through setting a different coefficient in the following formulars. The true odds ratio was ranged from 0.55 to 3.10 (-0.60 to 1.13 in log scale).

- Sensitivity 1 (true odds ratio = 3.10): $logit(Y_b) = 2.5 - 0.05 * C - 1 * U + 2 * A + e$
- Sensitivity 2 (true odds ratio = 1.03): $logit(Y_b) = 2.5 - 0.05 * C - 1 * U + 0 * A + e$
- Sensitivity 3 (true odds ratio = 0.55): $logit(Y_b) = 2.5 - 0.05 * C - 1 * U - 1 * A + e$

Performance of different methods for estimating the treatment effects of binary outcome in scenario 3 (n=200)

Treatment effect for Y_b /Method	Scenario 3 (one large unblocked confounding path)			
	Bias	RMSE	Coverage	Width
**Log(odds ratio) = 1.13**				
Raw	-0.459	0.545	0.652	1.156
GC	-0.147	0.375	0.940	1.386
PS_IPTW	-0.171	0.465	0.811	1.177
PS_OW	-0.140	0.392	0.863	1.173
PS_SMR	-0.204	0.564	0.711	1.170
TMLE	-0.160	0.453	0.963	1.867
**Log(odds ratio) = 0.03**				
Raw	-0.489	0.568	0.623	1.150
GC	-0.185	0.394	0.925	1.391
PS_IPTW	-0.197	0.478	0.774	1.144
PS_OW	-0.184	0.405	0.845	1.138
PS_SMR	-0.167	0.544	0.726	1.169
TMLE	-0.182	0.461	0.962	1.859
**Log(odds ratio) = -0.60**				
Raw	-0.492	0.584	0.661	1.236
GC	-0.176	0.415	0.940	1.505
PS_IPTW	-0.251	0.522	0.758	1.215
PS_OW	-0.185	0.428	0.846	1.201
PS_SMR	-0.208	0.572	0.736	1.243
TMLE	-0.232	0.511	0.966	2.018

GC, g-computation; RMSE, root mean squared error; PS-, propensity score-based; IPTW, inverse probability of treatment weighting; SMR, standardized mortality or morbidity ratio; OW, overlap weighting; TMLE, targeted maximum likelihood estimation. Bias was the average difference between the true value (simulated) and its estimate across the simulation replicates using the log-transformed scale for the binary outcome, such as log(odds ratio). RMSE was the square root of the mean squared error (MSE) that is the average squared difference between the true value and its estimate across the simulation replicates. Coverage was the proportion of times the 95% confidence interval of the estimate contained the true value. Width was the average difference between the upper and lower bounds of 95% confidence interval of estimate.

A similar sensitivity analysis was also conducted in scenario 1 and scenario 2 (see below).

Performance of different methods for estimating the treatment effects of binary outcome in scenario 1 (n=200)

**Treatment effect for Y_b /Method**	**Scenario 1 (small and blocked confounding paths)**			
	Bias	RMSE	Coverage	Width
**Log(odds ratio) = 1.18**				
Raw	-0.249	0.394	0.864	1.207
GC	0.035	0.363	0.952	1.433
PS_IPTW	0.040	0.439	0.838	1.217
PS_OW	0.052	0.389	0.884	1.206
PS_SMR	0.039	0.514	0.761	1.199
TMLE	0.048	0.438	0.976	1.948
**Log(odds ratio) = 0.01**				
Raw	-0.257	0.390	0.859	1.146
GC	-0.009	0.356	0.942	1.381
PS_IPTW	-0.017	0.426	0.823	1.132
PS_OW	-0.001	0.368	0.875	1.128
PS_SMR	-0.029	0.502	0.754	1.146
TMLE	-0.003	0.420	0.970	1.836
**Log(odds ratio) = -0.58**				
Raw	-0.279	0.422	0.855	1.209
GC	-0.026	0.372	0.949	1.460
PS_IPTW	-0.074	0.465	0.805	1.179
PS_OW	-0.026	0.383	0.884	1.172
PS_SMR	-0.023	0.524	0.751	1.199
TMLE	-0.051	0.460	0.968	1.960

GC, g-computation; RMSE, root mean squared error; PS-, propensity score-based; IPTW, inverse probability of treatment weighting; SMR, standardized mortality or morbidity ratio; OW, overlap weighting; TMLE, targeted maximum likelihood estimation. Bias was the average difference between the true value (simulated) and its estimate across the simulation replicates using the log-transformed scale for the binary outcome, such as log(odds ratio). RMSE was the square root of the mean squared error (MSE) that is the average squared difference between the true value and its estimate across the simulation replicates. Coverage was the proportion of times the 95% confidence interval of the estimate contained the true value. Width was the average difference between the upper and lower bounds of 95% confidence interval of estimate.

Performance of different methods for estimating the treatment effects of binary outcome in scenario 2 (n=200)

**Treatment effect for Y_b /Method**	**Scenario 2 (medium and blocked confounding paths)**			
	Bias	RMSE	Coverage	Width
**Log(odds ratio) = 1.13**				
Raw	-0.318	0.436	0.802	1.174
GC	0.015	0.348	0.951	1.382
PS_IPTW	-0.005	0.442	0.832	1.190
PS_OW	0.031	0.374	0.887	1.185
PS_SMR	0.001	0.506	0.753	1.182
TMLE	0.011	0.431	0.972	1.865
**Log(odds ratio) = 0.04**				
Raw	-0.343	0.451	0.795	1.154
GC	-0.005	0.348	0.946	1.377
PS_IPTW	-0.008	0.430	0.820	1.141
PS_OW	0.004	0.362	0.886	1.138
PS_SMR	0.003	0.487	0.784	1.168
TMLE	0.009	0.417	0.971	1.829
**Log(odds ratio) = -0.58**				
Raw	-0.345	0.470	0.813	1.233
GC	0.007	0.376	0.949	1.478
PS_IPTW	-0.068	0.478	0.806	1.200
PS_OW	0.007	0.387	0.878	1.190
PS_SMR	-0.027	0.515	0.790	1.232
TMLE	-0.039	0.469	0.969	1.984

GC, g-computation; RMSE, root mean squared error; PS-, propensity score-based; IPTW, inverse probability of treatment weighting; SMR, standardized mortality or morbidity ratio; OW, overlap weighting; TMLE, targeted maximum likelihood estimation. Bias was the average difference between the true value (simulated) and its estimate across the simulation replicates using the log-transformed scale for the binary outcome, such as log(odds ratio). RMSE was the square root of the mean squared error (MSE) that is the average squared difference between the true value and its estimate across the simulation replicates. Coverage was the proportion of times the 95% confidence interval of the estimate contained the true value. Width was the average difference between the upper and lower bounds of 95% confidence interval of estimate.
