# Supplementary material for: Comparing g-computation, propensity score-based weighting, and targeted maximum likelihood estimation for analyzing externally controlled trials with both measured and unmeasured confounders: a simulation study
Source: BMC Med Res Methodol. 2023 Jan 17;23:18. doi: 10.1186/s12874-023-01835-6 (PMC9843888; doi:10.1186/s12874-023-01835-6)
Supplement: Supplementary file 3 — Additional file 3: Supplementary file S3. R programming example for the simulation. [file 12874_2023_1835_MOESM3_ESM.pdf]

```
# Supplementary file S3 (R codes example)

##########################################################################################
# G-computation versus propensity score-based methods
#
# Establish a comparable control arm for a single-arm clinical trial
# (Cope with baseline covariates only)
#-----------------------------------------------------------------------------------
#Data set
# C: fixed baseline covariate, e.g. age
# L0: time-varying covariate at baseline, e.g. ECOG performance status 0-1 vs 2+
# A0: time-varying covariate at baseline, e.g. treatment
# Ya: continuous outcome, e.g. mobility score
# Yb: binary outcome, e.g. treatment response
# Yc: time-to-event outcome, e.g. overall survival
# D: randomly censoring outcome, e.g. 1-Death
##########################################################################################

library(broom) # for making model summary tidy
library(simstudy) # for simulation data set
library(tidyverse) # multiple packages for visualize and manage data e.g. ggplot2, dplyr
library(RISCA) # for g-computation with binary and time to event outcomes
library(gfoRmula) # for g-computation for time-varying treatment
library(plotly) # to plot interactive graphs
library(simhelpers) # to summarize the simulation results
library(kableExtra) # to create a kable table
library(data.table) # for data manipulation operations
library(SuperLearner) # to utilize super learning for estimation
library(ltmle) # longitudinal Targeted Maximum Likelihood Estimation (doubly robust method)


#-------------------------------------------------
#Step 1: Create a data set (with treatment effect)
#consider as a target population
#-------------------------------------------------

set.seed(123)
defMSM<- defData(varname = "U", formula = 0.5, variance = 1, dist = "normal")

defMSM<- defData(defMSM, varname = "C", formula = 50, variance = 50, dist = "normal")

defMSM<- defData(defMSM, varname = "e0", formula = 0, variance = 2, dist = "normal")
defMSM<- defData(defMSM, varname = "L0", formula = "-2.66+ U*2 + e0",
            dist = "binary", link = "logit")

defMSM<- defData(defMSM, varname = "e1", formula = 0, variance = 1, dist = "normal")
defMSM<- defData(defMSM, varname = "A0", formula = "-13.42 + L0*1.5 + C*0.25 + e1",
            dist = "binary", link = "logit")

defMSM<- defData(defMSM, varname = "e2", formula = 0, variance = 4, dist = "normal")
defMSM<- defData(defMSM, varname = "Ya",
            formula = "50 - C*0.1 - U*1.5 + A0*4 + e2",
            dist = "nonrandom")
```

```
defMSM<- defData(defMSM, varname = "e3", formula = 0, variance = 4, dist = "normal")
defMSM<- defData(defMSM, varname = "Yb",
                formula = "2.5 - C*0.05 - U*1 + A0*2  + e3",
                dist = "binary", link = "logit")

defMSM<- defData(defMSM, varname = "X", formula = "0;1", dist = "uniform")
defMSM<- defData(defMSM, varname = "e4", formula = 0, variance = 0.4, dist = "normal")
defMSM<- defData(defMSM, varname = "Yc",
                formula = "(-log(X)*exp(5.0 - C*0.03 - U*0.25 - A0*log(1/3) + e4))^(1/1.2)",
                dist = "nonrandom")
defMSM<- defData(defMSM, varname = "D",
                formula = 0.8,
                dist = "binary")

defMSM
dt <- genData(20000, defMSM)


#---------------------------------
#Step 2: set up the true effect
#---------------------------------

# Mean difference
fit_ya <- glm(Ya ~ A0 + L0 + U + C, data=dt)
true_ya =  matrix(fit_ya$coefficients)[2,1]

# Odds Ratio
fit_yb<- glm(Yb ~ A0 + L0 + U + C, family=quasibinomial("logit"), data=dt)
gc.yb<- gc.logistic(glm.obj=fit_yb, data=dt, group="A0", effect="ATE",
                   var.method='bootstrap', iterations=2, n.cluster=1)
true_yb = gc.yb$logOR[,1]

# Hazard ratio
fit_yc<- coxph(Surv(Yc, D) ~ A0 + U + C,  data=dt)
gc.yc<- gc.survival(coxph.obj=fit_yc, data=dt, group="A0", time='Yc', failure='D', effect="ATE",
                   max.time=max(dt$Yc), iterations=2, n.cluster=1)
true_yc = gc.yc$logHR[,1]


#---------------------------------
#Step 3: sampling and calculation
#---------------------------------
#Initial value
n_sim = 3000
n_patients = 200
n_methods = 7
output_ya = data.frame(matrix(data=NA, n_sim*n_methods, 4))
output_yb = data.frame(matrix(data=NA, n_sim*n_methods, 4))
output_yc = data.frame(matrix(data=NA, n_sim*n_methods, 4))
colnames(output_ya)<- c("Model", "A0_est", "A0_lower", "A0_upper")
colnames(output_yb)<- c("Model", "A0_est", "A0_lower", "A0_upper")
colnames(output_yc)<- c("Model", "A0_est", "A0_lower", "A0_upper")
for (i in 1:n_sim){
```

```
sim = dt[dt$id %in% sample(dt$id, n_patients, replace = F),]
  #Method 1: Multivariable linear regression
  fit_ya = glm(Ya ~ A0 + L0 + C, data = sim)
    output_ya[1+n_methods*(i-1), 1] = "MLR"
    output_ya[1+n_methods*(i-1), 2] =  fit_ya$coefficients[2]
    output_ya[1+n_methods*(i-1), 3:4] = confint.default(fit_ya)[2, 1:2]
  fit_yb = glm(Yb ~ A0 + L0 + C , family=quasibinomial("logit"), data = sim)
    output_yb[1+n_methods*(i-1), 1] = "MLR"
    output_yb[1+n_methods*(i-1), 2] =  fit_yb$coefficients[2]
    output_yb[1+n_methods*(i-1), 3:4] = confint.default(fit_yb)[2, 1:2]
  fit_yc = coxph(Surv(Yc, D) ~ A0 + L0 + C, data = sim)
    output_yc[1+n_methods*(i-1), 1] = "MLR"
    output_yc[1+n_methods*(i-1), 2] =  fit_yc$coefficients[1]
    output_yc[1+n_methods*(i-1), 3:4] = confint.default(fit_yc)[1, 1:2]

  #Method 2: Propensity score-based
    # function to convert propensity scores to IPW
    fitA0 <- glm(A0 ~ L0 + C, data = sim, family=binomial)
    sim[, predA0 := predict(fitA0, type = "response")]
    getWeight <- function(predA0, actA0, method) {
      if(method==1){
          wt <- 1/ (actA0*predA0 + (1-actA0)*(1-predA0)) # IPTW
        }else if(method==2){
          wt <- actA0*(1-predA0) + (1-actA0)*predA0      # Overlapping weighting
        }else if(method==3){
          wt <- actA0*1 + (1-actA0)*predA0/(1-predA0)    # SMR
        }
      return(wt)
    }
    sim[, iptw:= getWeight(predA0, A0, 1)]
    sim[, ow:= getWeight(predA0, A0, 2)]
    sim[, smr:= getWeight(predA0, A0, 3)]
    sim=data.table(sim)

    #PS-IPTW
    fit_ya = glm(Ya ~ A0 , weights = iptw, data = sim)
      output_ya[2+n_methods*(i-1), 1] = "PS_IPTW"
      output_ya[2+n_methods*(i-1), 2] =  fit_ya$coefficients[2]
      output_ya[2+n_methods*(i-1), 3:4] = confint.default(fit_ya)[2, 1:2]
    fit_yb = glm(Yb ~ A0 , family=quasibinomial("logit"), weights = iptw, data = sim)
      output_yb[2+n_methods*(i-1), 1] = "PS_IPTW"
      output_yb[2+n_methods*(i-1), 2] =  fit_yb$coefficients[2]
      output_yb[2+n_methods*(i-1), 3:4] = confint.default(fit_yb)[2, 1:2]
    fit_yc = coxph(Surv(Yc, D) ~ A0,  weights = iptw, data = sim)
      output_yc[2+n_methods*(i-1), 1] = "PS_IPTW"
      output_yc[2+n_methods*(i-1), 2] =  fit_yc$coefficients[1]
      output_yc[2+n_methods*(i-1), 3:4] = confint.default(fit_yc)[1, 1:2]

    #PS-OW
    fit_ya = glm(Ya ~ A0 , weights = ow, data = sim)
      output_ya[3+n_methods*(i-1), 1] = "PS_OW"
      output_ya[3+n_methods*(i-1), 2] =  fit_ya$coefficients[2]
      output_ya[3+n_methods*(i-1), 3:4] = confint.default(fit_ya)[2, 1:2]
    fit_yb = glm(Yb ~ A0 , family=quasibinomial("logit"), weights = ow, data = sim)
```

```
      output_yb[3+n_methods*(i-1), 1] = "PS_OW"
      output_yb[3+n_methods*(i-1), 2] =  fit_yb$coefficients[2]
      output_yb[3+n_methods*(i-1), 3:4] = confint.default(fit_yb)[2, 1:2]
    fit_yc = coxph(Surv(Yc, D) ~ A0,  weights = ow, data = sim)
      output_yc[3+n_methods*(i-1), 1] = "PS_OW"
      output_yc[3+n_methods*(i-1), 2] =  fit_yc$coefficients[1]
      output_yc[3+n_methods*(i-1), 3:4] = confint.default(fit_yc)[1, 1:2]

    #PS-SMR
    fit_ya = glm(Ya ~ A0 , weights = smr, data = sim)
      output_ya[4+n_methods*(i-1), 1] = "PS_SMR"
      output_ya[4+n_methods*(i-1), 2] =  fit_ya$coefficients[2]
      output_ya[4+n_methods*(i-1), 3:4] = confint.default(fit_ya)[2, 1:2]
    fit_yb = glm(Yb ~ A0 , family=quasibinomial("logit"), weights = smr, data = sim)
      output_yb[4+n_methods*(i-1), 1] = "PS_SMR"
      output_yb[4+n_methods*(i-1), 2] =  fit_yb$coefficients[2]
      output_yb[4+n_methods*(i-1), 3:4] = confint.default(fit_yb)[2, 1:2]
    fit_yc = coxph(Surv(Yc, D) ~ A0,  weights = smr, data = sim)
      output_yc[4+n_methods*(i-1), 1] = "PS_SMR"
      output_yc[4+n_methods*(i-1), 2] =  fit_yc$coefficients[1]
      output_yc[4+n_methods*(i-1), 3:4] = confint.default(fit_yc)[1, 1:2]

  #Method 3: G-computation
    #Bootstrap (re-sampling) for Ya
    ya_boots = matrix(NA, 100, 1)
    for (j in 1:100) {
      id <- sort(sample(unique(sim$id), 1*length(unique(sim$id)), replace=T)) #Bootstrap (with replacement)
      id <- data.frame(cbind(id, IDNEW=c(1:length(id))))
      sim_ya<- merge(sim, id, by="id")

      fit_ya<- glm(Ya ~ A0 + L0 + C, data = sim_ya)
      sim1 = sim_ya
      sim1$A0 = 1
      sim0 = sim_ya
      sim0$A0 = 0
      ya_boots[j, 1] = mean(predict(fit_ya, sim1)) - mean(predict(fit_ya, sim0))
    }
      output_ya[5+n_methods*(i-1), 1] = "GC"
      output_ya[5+n_methods*(i-1), 2] =  mean(ya_boots)
      output_ya[5+n_methods*(i-1), 3] =  mean(ya_boots) - qnorm(0.975)*sqrt(cov(ya_boots))
      output_ya[5+n_methods*(i-1), 4] =  mean(ya_boots) + qnorm(0.975)*sqrt(cov(ya_boots))

    fit_yb<- glm(Yb ~ A0 + L0 + C, family=quasibinomial("logit"), data = sim)
    gc.yb<- gc.logistic(glm.obj=fit_yb, data = sim, group="A0", effect="ATE",
                      var.method='bootstrap', iterations=100, n.cluster=1)
      output_yb[5+n_methods*(i-1), 1] = "GC"
      output_yb[5+n_methods*(i-1), 2] = gc.yb$logOR[,1]
      output_yb[5+n_methods*(i-1), 3:4] = gc.yb$logOR[,3:4]

    fit_yc<- coxph(Surv(Yc, D) ~ A0 + L0 + C,  data = sim)
    gc.yc<- gc.survival(coxph.obj=fit_yc, data = sim, group="A0", time='Yc', failure='D', effect="ATE",
                      max.time=max(sim$Yc), iterations=100, n.cluster=1)
      output_yc[5+n_methods*(i-1), 1] = "GC"
      output_yc[5+n_methods*(i-1), 2] = gc.yc$logHR[,1]
```

```
      output_yc[5+n_methods*(i-1), 3:4] = gc.yc$logHR[,3:4]

   #Method 4: Doubly robust estimator (TMLE)
      #Treatment at baseline: A0
      Anodes = c("A0")
      #Censoring indicator: D
      Cnodes = c("Censor")
      #Outcome: Y
      Ynodes = c("Y")

     sim_ya <- data.frame(W1=sim$C, W2=sim$L0, A0=sim$A0, Y=sim$Ya)
     suppressWarnings(suppressMessages(
      fit_ya <- ltmle(sim_ya, Anodes = Anodes, Lnodes = NULL, Ynodes = Ynodes, abar =list(1,0), SL.library =
"default", gcomp = F)
     ))
      output_ya[6+n_methods*(i-1), 1] = "TMLE"
      output_ya[6+n_methods*(i-1), 2] =  summary(fit_ya)[["effect.measures"]][["ATE"]][["estimate"]]
      output_ya[6+n_methods*(i-1), 3:4] =  summary(fit_ya)[["effect.measures"]][["ATE"]][["CI"]]

     sim_yb <- data.frame(W1=sim$C, W2=sim$L0, A0=sim$A0, Y=sim$Yb)
     suppressWarnings(suppressMessages(
      fit_yb <- ltmle(sim_yb, Anodes = Anodes, Lnodes = NULL, Ynodes = Ynodes, abar =list(1,0), SL.library =
'default', gcomp = F)
     ))
      output_yb[6+n_methods*(i-1), 1] = "TMLE"
      output_yb[6+n_methods*(i-1), 2] =  log(summary(fit_yb)[["effect.measures"]][["OR"]][["estimate"]])
      output_yb[6+n_methods*(i-1), 3:4] =  log(summary(fit_yb)[["effect.measures"]][["OR"]][["CI"]])

     sim_yc <- data.frame(W1=sim$C, W2=sim$L0, A0=sim$A0, Censor=sim$D)
     sim_yc$Y1 = ifelse(sim$Yc<=22 & sim_yc$Censor==1, 1, 0)  #create a new outcome at the primary time point (e.g.
median survival).
     Ynodes = c("Y1")
     suppressWarnings(suppressMessages(
      fit_yc <- ltmle(sim_yc, Anodes = Anodes, Lnodes = NULL, Cnodes = Cnodes, Ynodes = Ynodes, abar =list(1,0),
              survivalOutcome=T, SL.library = 'default', gcomp = F)
     ))
      output_yc[6+n_methods*(i-1), 1] = "TMLE"
      output_yc[6+n_methods*(i-1), 2] = log(summary(fit_yc)[["effect.measures"]][["RR"]][["estimate"]]) #Use RR to
estimate HR
      output_yc[6+n_methods*(i-1), 3:4] = log(summary(fit_yc)[["effect.measures"]][["RR"]][["CI"]])

   #Method 5: Unadjusted (raw)
     fit_ya = glm(Ya ~ A0, data = sim)
      output_ya[7+n_methods*(i-1), 1] = "Raw"
      output_ya[7+n_methods*(i-1), 2] =  fit_ya$coefficients[2]
      output_ya[7+n_methods*(i-1), 3:4] =  confint.default(fit_ya)[2, 1:2]
     fit_yb = glm(Yb ~ A0 , family=quasibinomial("logit"), data = sim)
      output_yb[7+n_methods*(i-1), 1] = "Raw"
      output_yb[7+n_methods*(i-1), 2] =  fit_yb$coefficients[2]
      output_yb[7+n_methods*(i-1), 3:4] =  confint.default(fit_yb)[2, 1:2]
     fit_yc = coxph(Surv(Yc, D) ~ A0, data = sim)
      output_yc[7+n_methods*(i-1), 1] = "Raw"
      output_yc[7+n_methods*(i-1), 2] =  fit_yc$coefficients[1]
      output_yc[7+n_methods*(i-1), 3:4] =  confint.default(fit_yc)[1, 1:2]
```

```
}


output_ya = cbind(output_ya, true_ya)
output_yb = cbind(output_yb, true_yb)
output_yc = cbind(output_yc, true_yc)

#--------------------------------
#Step 4: Plot and summary
#--------------------------------

#Convert RR to HR for yc
output_yc$A0_est = ifelse(output_yc$Model=="TMLE",
                          log(log(1-0.4*exp(output_yc$A0_est))/log(1-0.4)),
                          output_yc$A0_est)

output_yc$A0_lower = ifelse(output_yc$Model=="TMLE",
                            log(log(1-0.4*exp(output_yc$A0_lower))/log(1-0.4)),
                            output_yc$A0_lower)

output_yc$A0_upper = ifelse(output_yc$Model=="TMLE",
                            log(log(1-0.4*exp(output_yc$A0_upper))/log(1-0.4)),
                            output_yc$A0_upper)

#Plot
ggplotly(ggplot(output_ya, aes(x=A0_est, color=Model))
         + geom_density(aes(color=Model))
         + ylab("Density")+xlab("Estimate of effect: mean difference")
         + geom_vline(xintercept = true_ya)+theme_bw()
)
ggplot(output_ya[output_ya$Model!="MLR",], aes(x=A0_est, color=Model)) +
  geom_density(aes(color=Model), size=1) +
  ylab("Density")+xlab("Estimate of effect: mean difference") +
  geom_vline(xintercept = true_ya)+theme_bw() +
  scale_x_continuous(limits=c(1.5,6)) +
  geom_text(aes(x = 4.4, y = 1.1,
                label = "True effect"), size=4, colour="gray35")+
  geom_segment(aes(x=4.5, y=0.75, xend=6, yend=0.75), arrow=arrow(length=unit(0.2, "cm")),size=1.5,
colour="gray60")+
  geom_text(aes(x = 5.2, y = 0.78, label = "Favors treatment"), size=4,colour="gray40") +
  scale_color_manual(values=c("red", "green", "blue", "purple", "yellow3", "black"))

ggplotly(ggplot(output_yb, aes(x=A0_est, color=Model))
         + geom_density()
         + ylab("Density")+xlab("Estimate of effect: log(odds ratio)")
         + geom_vline(xintercept = true_yb)+theme_bw()
         + scale_x_continuous(limits=c(-1,3))
         + geom_text(aes(x = 1.4, y = 1.5,
                         label = "True effect"), size=3.5, colour="gray35")
)
ggplot(output_yb[output_yb$Model!="MLR",], aes(x=A0_est, color=Model)) +
  geom_density(aes(color=Model), size=1) +
  ylab("Density")+xlab("Estimate of effect: log(odds ratio)") +
  geom_vline(xintercept = true_yb)+theme_bw() +
```

```
  geom_vline(xintercept = 0, colour="gray40", linetype="dashed")+theme_bw() +
  scale_x_continuous(limits=c(0,3.5)) +
  geom_text(aes(x = 2.1, y = 1.3,
               label = "True effect"), size=4, colour="gray35")+
  geom_segment(aes(x=0.02, y=1.45, xend=1, yend=1.45), arrow=arrow(length=unit(0.2, "cm")),size=1.5,
colour="gray60")+
  geom_text(aes(x = 0.5, y = 1.5, label = "Favors treatment"), size=4,colour="gray40") +
  scale_color_manual(values=c("red", "green", "blue", "purple", "yellow3", "black"))

ggplotly(ggplot(output_yc, aes(x=A0_est, color=Model))
          + geom_density()
          + ylab("Density")+xlab("Estimate of effect: log(hazard ratio)")
          + geom_vline(xintercept = true_yc)+theme_bw()
        # + scale_x_continuous(limits=c(-1,3))
)
ggplot(output_yc[output_yc$Model!="MLR",], aes(x=A0_est, color=Model)) +
  geom_density(aes(color=Model), size=1) +
  ylab("Density")+xlab("Estimate of effect: log(hazard ratio)") +
  geom_vline(xintercept = true_yc)+theme_bw() +
  geom_vline(xintercept = 0, colour="gray40", linetype="dashed")+theme_bw() +
  scale_x_continuous(limits=c(-2.0, 0)) +
  geom_text(aes(x = -0.9, y = 2.5,
               label = "True effect"), size=4, colour="gray35")+
  geom_segment(aes(x=-0.02, y=2.75, xend=-0.5, yend=2.75), arrow=arrow(length=unit(0.2, "cm")),size=1.5,
colour="gray60")+
  geom_text(aes(x = -0.25, y = 2.85, label = "Favors treatment"), size=4,colour="gray40") +
  scale_color_manual(values=c("red", "green", "blue", "purple", "yellow3", "black"))



#Summarize the simulation results

k_ya<- output_ya %>%
  group_by(Model) %>%
  do(calc_absolute(., estimates = A0_est, true_param = true_ya))
c_ya<- output_ya %>%
  group_by(Model) %>%
  do(calc_coverage(., lower_bound = A0_lower, upper_bound = A0_upper, true_param = true_ya))
kc_ya<- data.frame(Model=k_ya$Model, K=k_ya$K, bias=k_ya$bias,
                   var=k_ya$var, mse=k_ya$mse, rmse=k_ya$rmse, coverage=c_ya$coverage, width=c_ya$width)
kc_ya<-kc_ya %>%
  kable(digits =5, format="simple") #create a kable table
print(kc_ya)

k_yb<- output_yb %>%
  group_by(Model) %>%
  do(calc_absolute(., estimates = A0_est, true_param = true_yb))
c_yb<- output_yb %>%
  group_by(Model) %>%
  do(calc_coverage(., lower_bound = A0_lower, upper_bound = A0_upper, true_param = true_yb))
kc_yb<- data.frame(Model=k_yb$Model, K=k_yb$K, bias=k_yb$bias,
                   var=k_yb$var, mse=k_yb$mse, rmse=k_yb$rmse, coverage=c_yb$coverage, width=c_yb$width)
kc_yb<-kc_yb %>%
  kable(digits =5, format="simple") #create a kable table
```

```
print(kc_yb)


k_yc<- output_yc %>%
  group_by(Model) %>%
  do(calc_absolute(., estimates = A0_est, true_param = true_yc))
c_yc<- output_yc %>%
  group_by(Model) %>%
  do(calc_coverage(., lower_bound = A0_lower, upper_bound = A0_upper, true_param = true_yc))
kc_yc<- data.frame(Model=k_yc$Model, K=k_yc$K, bias=k_yc$bias,
                   var=k_yc$var, mse=k_yc$mse, rmse=k_yc$rmse, coverage=c_yc$coverage, width=c_yc$width)
kc_yc<-kc_yc %>%
  kable(digits =5, format="simple") #create a kable table
print(kc_yc)
```
